# Human Papillomaviruses in Adolescents: Knowledge, Attitudes, and Practices of Pharmacists Regarding Virus and Vaccination in France

**DOI:** 10.3390/v15030778

**Published:** 2023-03-17

**Authors:** Lucas Dufour, Florence Carrouel, Claude Dussart

**Affiliations:** 1 Laboratory “Health, Systemic, Process” (P2S), UR4129, University Claude Bernard Lyon 1, University of Lyon, 69008 Lyon, Franceflorence.carrouel@univ-lyon1.fr (F.C.); 2 Hospices Civils of Lyon, 69003 Lyon, France

**Keywords:** Human papillomavirus (HPV), boy, vaccination hesitancy, France, pharmacists, pharmacy practice, prevention

## Abstract

*Human papillomaviruses* (HPVs) are responsible for one of the most common sexually transmitted diseases in the world, and their oncogenic role has been well demonstrated in genital, anal, and oropharyngeal areas. However, a certain distrust and a lack of knowledge about this vaccine are perceptible among French adolescents and their parents. Thus, health professionals and, more particularly, pharmacists appear to be key persons to promote HPV vaccination and restore confidence in the target population. The present study aims to assess the knowledge, attitudes, and practices regarding HPV vaccination among pharmacists, particularly in boys, following the 2019 recommendation to vaccinate them. The present study was designed as a cross-sectional, quantitative, and descriptive survey that was conducted from March to September 2021 among pharmacists in France. 215 complete questionnaires were collected. Gaps in knowledge were found, only 21.4% and 8.4% obtained a high level of knowledge related to, respectively, HPV and vaccination. Pharmacists were confident in the HPV vaccine (94.4%), found it safe and useful, and felt that the promotion of the vaccine was part of their role (94.0%). However, only a few have already advised it, which they justify due to a lack of opportunity and forgetfulness. Faced with this, training, computerized reminders, or supportive materials could be implemented to improve the advice and thus the vaccination coverage. Finally, 64.2% were in favor of a pharmacy-based vaccination program. In conclusion, pharmacists are interested in this vaccination and the role of promoter. However, they need the means to facilitate this mission: training, computer alerts, supportive materials such as flyers, and the implementation of vaccination in pharmacies.

## 1. Introduction

*Human papillomaviruses* (HPVs) are responsible for one of the most common sexually transmitted diseases world. HPV infections are usually subclinical and do not last more than two years. Benign manifestations are usually observed, but it has been established that persistent HPV infection is associated with cervical, anogenital, and head and neck cancers [1]. One of the key events in HPV-induced carcinogenesis is the integration of the HPV genome into a host chromosome; other similar events are also observed [2,3,4]. Currently, more than 200 HPV types have been identified [5] and organized into five main genera (alpha, beta, gamma, mu, and nu) [6]. HPVs have different oncogenic potentials and can be responsible for three types of HPV infections: non-genital (cutaneous), mucosal, or anogenital and epidermodysplasia verruciformis [7]. Regarding the mucosal HPV, there are high-risk/oncogenic HPV types, which can be potentially carcinogenic (HPV16, 18, 31, and 33), and low-risk/nononcogenic HPV types (HPV6 and 11), which are mainly found in warts [8].

**Figure 1:**
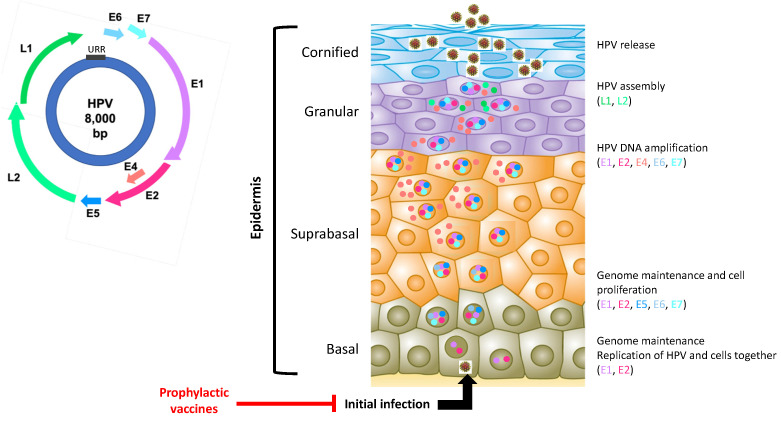
Human papillomavirus genome organization and viral life cycle. The HPV genome is composed of the early viral genes (E1, E2, E4, E5, E6, E7), the late viral genes (L1 and L2), and the upstream regulatory region (URR). The HPV life cycle is composed of several steps. First, the virus infects a keratinocyte of the basal layer of the epithelium. Second, the virus and the cell replicate together. As long as the cell is dividing, the expression of the viral genes is highly controlled. Third, when the cell stops dividing and begins to differentiate into a mature keratinocyte, the virus activates all its genes. The expression of the oncogenic genes is no longer regulated. Finally, in the upper layers of the epithelium, all viral genes are expressed, and the viral genomes are encapsidated. The encapsidated viral particles leave the cell.

The genetic material consists of several open reading frames encoding proteins involved in viral DNA replication (E1 and E2), regulation of viral gene expression (E2), virion formation (E4), and cell immortalization and transformation (E5, E6, E7) for high-risk HPV only [9,10] (Figure 1). The L1 and L2 open reading frames encode capsid proteins. HPVs infect epithelial stem cells. A complete HPV multiplication cycle consists of three phases that, following sequential expression of viral genes, allow replication of viral DNA and subsequent production of new infectious virions [11]. The integration of viral DNA only concerns high-risk HPV. This leads to an overexpression of the two viral oncoproteins E6 and E7 which interfere with critical cell-cycle points, such as suppressive tumor protein p53 and retinoblastoma protein pRB. The action of E6 and E7 combined with that of E5 promotes cell immortalization and transformation [10,12,13].

In France, HPV-induced cancers accounted for 1.8% of incident cancers, or 6300 cases in 2015 [14]. The majority of these cases were cervical cancers (44%) or male cancers (nearly 30%) affecting five locations (oropharynx (1060 cases), anus (360 cases), oral cavity (100 cases), larynx (100 cases), and penis (100 cases)) [15]. In addition, the annual incidence of condyloma in men aged 20 to 30 years in France is estimated at 528 per 100,000, which corresponds to more than 23,000 new cases each year [16]. The French High Council of Public Health (*Haute Autorité de Santé*, HAS) estimates that 100,000 individuals are affected by condyloma each year in France [17].

HPV vaccines have been successfully implemented against up to nine HPV types, demonstrating high protection against cervical infections due to these HPV types as well as against condyloma and some HPV-related cancers [18,19,20,21,22,23]. In France, since 2007, vaccination against HPV has been recommended for girls between 11 and 19 years old [24,25]. Nevertheless, 15 years after their introduction, vaccination coverage in France remains very low among young girls (only 24% of them fully vaccinated in 2018 [26]). In a meta-analysis, Drolet et al. found that in countries with less than 50% vaccination coverage for girls, there was evidence of reduced condyloma in young women under the age of 20, but no herd immunity in older women and men [27]. Moreover, several studies demonstrated that HPV was implicated in men’s cancer [28,29,30]. Thus, the HAS issued an opinion in February 2016 on the vaccination strategy for boys. In addition to extending vaccination to individuals up to 26 years of age for men who have sex with men (MSM), it concluded that the cost-effectiveness of universal vaccination is favorable if all HPV-related pathologies are considered and/or if vaccination coverage in boys is high when vaccination coverage in girls is low (<40%, as is the case in France) [17]. This led, in 2019, the HAS to recommend the vaccination of all adolescents aged from 11 to 14 years (with catch-up vaccination until 19 years old), regardless of their gender and sexual orientation [31].

In France, vaccination is mainly carried out by private practitioners (general practitioners and gynecologists), who have a good level of acceptance of the HPV vaccine. They play a major role in parents’ decisions to vaccinate their children against HPV [32]. Ninety percent of general practitioners had a favorable opinion [33], and more than 75% of young girls were in favor of this vaccination [34,35,36,37]. The main barriers reported by girls were a lack of knowledge about vaccination, the cost of the vaccine, fear of adverse events, and parental refusal [33]. The cost of the HPV vaccine is not a real barrier because the vaccine is reimbursed at 65% by the *Caisse Primaire d’Assurance Maladie* and complementary organizations usually complete the reimbursement. Lack of knowledge is the main obstacle to HPV vaccination because it leads to fear and refusal of HPV vaccination [38]. HPV vaccine hesitancy is thought to be related to several determinants, such as epidemiological factors (socioeconomic status and health care availability) [39,40] or maternal attitudes toward cervical cancer screening [41].

In addition to practitioners, pharmacies can play an important role in increasing HPV vaccination rates [42]. Pharmacies are strategic sites because most families, including those living in rural areas, have access to pharmacies. In addition, pharmacies are typically open for extended hours and on weekends. Pharmacists are therefore essential partners in vaccine delivery because their scope of practice has been expanded [10]. They have a critical role in public health because of their accessibility, ability to provide education, and patient acceptance [43,44,45]. In France, they have the *diplôme d’état de Docteur en Pharmacie* or doctor of pharmacy degree (PharmD) after six years of higher education [46]. Since 2020, they have received courses on HPV vaccination mentioning the recommendation for girls and boys. Thus, their qualifications and proximity to patients make pharmacists major players in the promotion of HPV vaccination. However, several studies conducted in North America concluded that pharmacists have an overall positive perception of HPV vaccination but a general lack of knowledge and information about HPV [42,47,48,49].

This study aims to assess the knowledge, attitudes, and practices concerning HPV vaccination among pharmacists in France, particularly in boys, following the expansion of the recommendation. The research question was, “What are the knowledge, attitudes, and practices of pharmacists in France regarding HPV vaccination, particularly for boys?” Our main hypothesis was that a lack of knowledge about HPV vaccination among pharmacists was one of the causes of poor attitudes toward promoting this vaccine. The results will allow us to better understand the pharmacist’s position in the promotion of HPV vaccination and strengthen this position through the proposal of actions adapted to their needs.

## 2. Materials and Methods

### 2.1. Study Design

The present study was designed as a cross-sectional, quantitative, and descriptive survey, which was conducted from March to September 2021. This research was performed in accordance with the Checklist for Reporting Of Survey Studies (CROSS) (Supplementary Table S1) [50].

A convenient sampling strategy was used. The sample was recruited thanks to the list of e-mail of pharmacists that accepted to be contacted and were provided by the faculty of pharmacy of Lyon (*Institut des Sciences Pharmaceutiques et Biologiques*, ISPB) and the Regional Union of Health Professionals of Pharmacists (*Union Régional des Profesionnels de Santé*, URPS) in the Auvergne-Rhône Alpes region. Therefore, 2897 pharmacists received an invitation to respond to our questionnaire by email through the URPS database, and about 100 were contacted directly or indirectly by word of mouth. Participants were able to access the questionnaire through a link to a website. The questionnaire was open, with a first broadcast, then a relaunch during the summer. No nominative data were collected to ensure the anonymity and confidentiality of the data.

### 2.2. Study Population

To be included in this survey, the participants had to be (i) at least 18 years old; (ii) pharmacists, pharmacy assistants, or pharmacy students; (iii) practicing their profession in the region in France; (iv) fluent in the French language; and (v) agree to fulfill the online questionnaire.

### 2.3. Sample Size

With a confidence level of 95%, a confidence interval of 7%, and an average of 50% for any unknown percentage of the questionnaire, and considering the total number of pharmacists listed by the URPS (2897 email addresses provided), it was necessary to include at least 196 subjects in this study.

### 2.4. Ethics

The study was conducted in accordance with the Declaration of Helsinki and the reference methodology MR-004 (N°2226244 v0, made on 5 May 2022) of the French National Commission on Information Technology and Liberties (CNIL).

The declaration of research objectives, the statement that participation was voluntary, and the voluntary return of the completed questionnaire constituted implied consent [51].

### 2.5. Questionnaire Design

**Figure 2:**
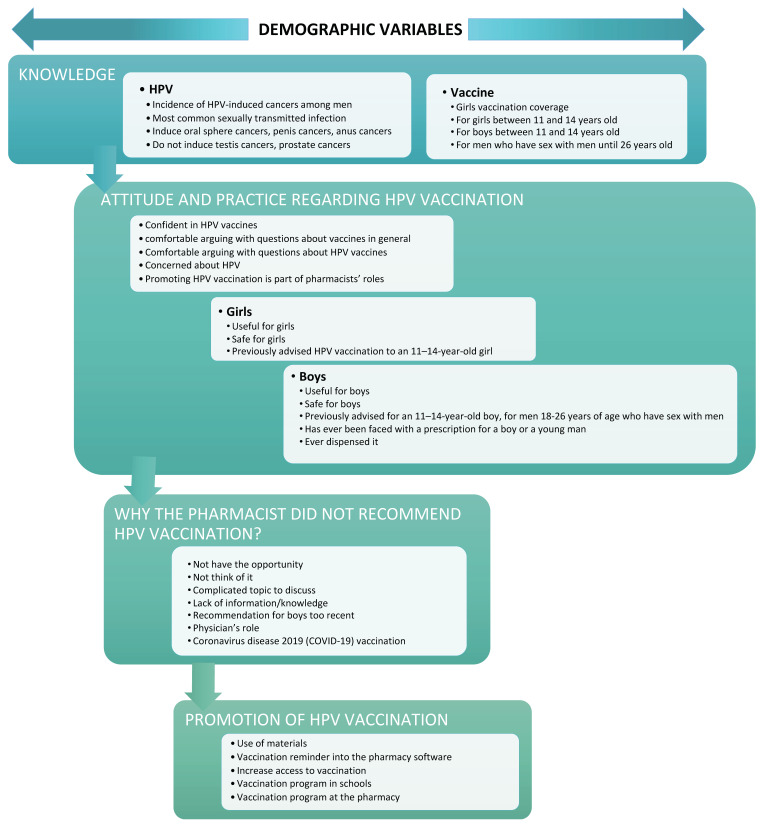
Conceptual framework of knowledge, attitudes, and practices as determinants for the promotion of HPV vaccination.

A structured survey to analyze knowledge, attitudes, and practices (KAP) regarding HPV and HPV vaccination, in the French language, was developed following the WHO recommendations [52]. This new survey instrument is based on the KAP conceptual framework presented in Figure 2.

The two principal investigators (L.D. and C.D.) conducted a literature review to identify published studies to evaluate knowledge and/or attitudes and/or practices regarding HPV and/or HPV vaccination. Based on the items found, questions were selected, adapted, or created to develop a preliminary questionnaire for pharmacists. For the validation of this questionnaire, we used the methodology described by Andrade et al. (2020) [53]. Both facial and content validation processes were used. Facial validation determines whether a questionnaire is able to do what it is intended to do, and content validation explores whether a questionnaire includes all necessary items, avoids unnecessary items, and is generally well-framed and well-presented. During the first step of validation, the questionnaire was analyzed by an Expert Committee composed of pharmacists and physicians specializing in HPV. They rated each item of the questionnaire as satisfactory or unsatisfactory on a validation sheet. If the expert rated one item as unsatisfactory, the reason was indicated, and improvements were suggested. The content validity index was calculated, and the questionnaire was revised considering the suggestions. The Expert Committee reviewed and approved this new version. During the second step of validation, this preliminary questionnaire was tested through cognitive debriefing methodology by 6 pharmacists and 4 students in their last years of pharmacy. The wording and comprehensiveness of the questionnaire were refined based on the participants’ perceptions and suggestions. This final version of the questionnaire was validated by the Expert Committee.

This questionnaire consisted of 46 questions, of which 39 were closed questions and 7 were open questions (Supplementary file Table S1). This questionnaire was composed of 3 parts (See Supplementary file): (i) sociodemographic data of the pharmacist and their pharmacy (5 questions), (ii) evaluation of knowledge of HPVs and vaccines (11 questions), and (iii) evaluation of attitude and practices regarding HPV vaccination (30 questions). This last part included 3 sections: (a) the pharmacist’s vision of vaccination and habits (12 questions), (b) promotional materials and aids (10 questions), and (c) the extension of recommendations and prospects for evolution (8 questions).

The questionnaire needed approximately 5 min to be completed. The questionnaire was accessible via an internet link that allowed an anonymous response. It was open from March to September 2021.

### 2.6. Data Analysis

From the collected responses, new variables were constituted:

- The levels of knowledge related to HPV using the responses of the 7 statements: “low” when 0 to 2 right answers were provided, “moderate” for 3 to 5 right answers, and “high” for 6 or 7 right answers;
- The levels of knowledge related to HPV vaccine were evaluated using the responses of the 4 statements: “low” when 0 or 1 right answer was provided, “moderate” for 2 or 3 right answers, and “high” for 4 right answers;
- The right attitude toward HPV vaccination in general corresponded to respondents that (i) were highly or very highly confident in HPV vaccines, (ii) felt comfortable or rather comfortable arguing with questions about vaccines in general, (iii) felt comfortable or rather comfortable arguing with questions about HPV vaccines, (iv) felt concerned about HPV, and (v) found that promoting HPV vaccination is part of pharmacists’ roles;
- The right attitude toward HPV vaccination in girls corresponded to respondents that (i) found the HPV vaccine useful for girls, (ii) considered that HPV vaccination was safe for girls, and (iii) had previously advised HPV vaccination to an 11–14-year-old girl;
- The right attitude toward HPV vaccination in boys corresponded to respondents that (i) found the HPV vaccine useful for boys, (ii) considered that HPV vaccination was safe for boys, and (iii) had previously advised HPV vaccination to an 11–14-year-old boy.

Data analysis was conducted using R software (version 4.2.1). The analysis was done according to the nature of the question. For closed questions, the descriptive analysis was directly presented by the percentages of responses to each item. For open-ended questions, we first listed all the answers sent in, and then we grouped the comments by association of ideas or keywords. We were thus able to quantify the qualitative variables by describing the frequencies obtained.

For the questions including a condition (for example, “if yes” to the previous question), we decided to analyze the questionnaire as it was elaborated and thus analyze only the answers answering this condition. A step of verification of the answers was thus carried out in order to check these conditions.

The percentages of respondents were rounded to the nearest value, with one decimal place.

Ordinal logistic regressions with cumulative link models were performed to evaluate the determinants of the level of knowledge of HPV and HPV vaccination. Covariates were gender, profession, number of years of practice, and the pharmacy typology.

Binary logistic regressions with generalized linear models were performed to evaluate the determinants of the right attitude toward HPV vaccination in general, toward HPV vaccination in girls, and toward HPV vaccination in boys, as well as the determinants of never having advised the vaccine.

Odds ratio with a 95% confidence interval and *p*-values were provided. A *p*-value below 0.05 was considered statistically significant.

## 3. Results

### 3.1. Sociodemographic Data of the Pharmacist and Their Pharmacy

In total, about 3000 pharmacists in the Auvergne-Rhône Alpes region were contacted (2897 through the URPS and about 100 through word of mouth) and 215 responses were collected, giving a response rate of about 7.2%.

The socio-demographic data of the participants are summarized in Table 1. The study population was mainly composed of pharmacy owners (75.8%, n = 163), women (67.4%, n = 145), those with between 11 and 20 years of experience (26.0%, n = 56) or between 21 and 30 years (26.5%, n = 57), and those practicing in rural (33.5%, n = 72) or neighborhood (35.3%, n = 76) pharmacies.

### 3.2. Knowledge of Pharmacists Regarding HPV and Its Vaccine

The data related to knowledge about HPV and its vaccine are summarized in Table 2 and Table S2. A total of 49.3% (n = 106) of respondents considered their level of knowledge as “excellent” or “rather excellent” concerning the virus, and 63.3% (n = 136) concerning the HPV vaccine. The level of knowledge related to HPV and its vaccination was high for, respectively, 21.4% (n = 46) and 8.4% (n = 18).

Concerning the incidence of HPV cancers in men in France, 26.0% (n = 56) estimated it to be less than 1000 cases/year, 42.3% (n = 91) between 1000 and 1500, 19.1% (n = 41) between 1500 and 2000, and 12.6% (n = 27) over 2000.

Among these cancers in men, 61.9% (n = 133) believed that they include oropharyngeal cancers, 60.9% (n = 131) penile cancers, 82.8% (n = 178) anal cancers, 32.6% (n = 70) testicular cancers, 16.3% (n = 35) prostate cancers. Added to the proposed answers were 0.5% (n = 1) genital warts, 0.5% (n = 1) “all genital system”, and 1.4% (n = 3) did not know.

Regarding the current recommendation of the vaccine, 99.5% (n = 214) of the respondents reported that it is recommended for girls between 11 and 14 years of age, with catch-up possible, and 89.3% (n = 192) that is recommended for boys at the same ages. However, concerning the recommendation for MSM, 22.8% (n = 49) thought that the vaccine is recommended up to 21 years of age, 34,4% (n = 74) up to 26 years of age, and 5.1% (n = 11) up to 30 years of age.

Of the respondents, 16.3% (n = 35) answered that girls’ vaccination coverage at the end of 2019 was less than 20%, 32.1% (n = 69) between 20 and 30%, 30.7% (n = 66) between 30 and 40%, 16.3% (n = 35) between 40 and 50%, and 4.7% (n = 10) more than 50%.

### 3.3. Pharmacists’ Perception and Habits

The third part of the questionnaire was divided into 3 subparts: the first one concerning the pharmacist’s perception of vaccination and habits is summarized in Table 3 and Table S3. Overall, the pharmacists were confident in the HPV vaccine (94.4% (n = 203)) and found it useful and safe for girls (respectively 99.5% (n = 214) and 92.6% (n = 199)) and for boys (respectively 92.1% (n = 201) and 87.9% (n = 192)).

Pharmacists felt concerned about HPV prevention (94.0% (n = 202)) and felt that promoting HPV vaccination was part of the role of the pharmacist (94.0% (n = 202)).

Concerning the advice regarding HPV vaccination, 69.8% (n = 150) said they had already advised a girl between 11 and 14 years of age, 34.0% (n = 73) a boy between 11 and 14 years of age, and 7.4% (n = 16) an MSM between 18 and 26 years of age. When they answered that they did not advise, the main reasons cited were the lack of opportunity (38.9%, n = 42) or the fact they did not think to do it (18.5%, n = 20). Other reasons cited were that it was complicated to discuss the subject (14.8%, n = 16), especially with parents or for MSM; lack of information/knowledge (14.8%, n = 16); the fact that the recommendations for boys were too recent (7.4%, n = 8); that it was the physician’s role (3.7%, n = 4); and the fact that the current battle was the vaccination against COVID-19 (1.9%, n = 2).

At the time of data collection, 70.7% (n = 152) of the respondents had ever received a prescription for an HPV vaccine for a boy or young man. Of these, 59.9% (n = 91) had dispensed and were already aware of the conditions for reimbursement.

Finally, the majority of pharmacists were comfortable or rather comfortable arguing in front of questions about vaccination in general (97.2% (n = 209)), as well as about HPV vaccination (79.1% (n = 170)).

### 3.4. Pharmacists’ Use and Interest in Materials and Aids for HPV Vaccination Promotion

**Figure 3:**
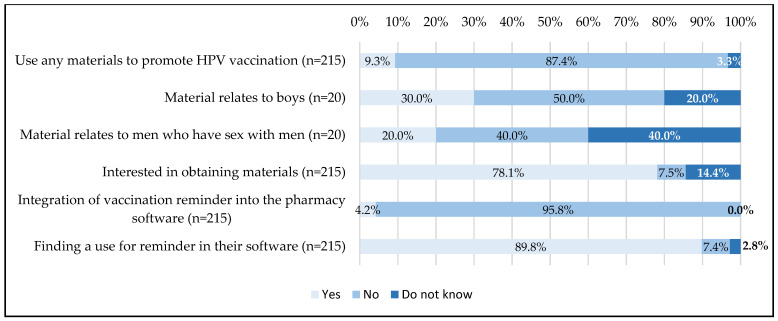
Pharmacists’ use and interest in materials and aids for HPV vaccination promotion.

The materials and aids used by pharmacists to promote HPV vaccination is presented in Figure 3.

87.4% (n = 188) did not use promotional materials but 78.1% (n = 168) of respondents would be interested in obtaining materials. In addition, of the 20 pharmacists who had materials, 30% (n = 6) reported that they referred to HPV vaccination of boys (11–14 years) and 20% (n = 4) that they addressed HPV vaccination of MSM.

Software that includes a reminder for vaccination was available in 4.2% (n = 9) of pharmacies but 89.8% (n = 193) of the respondents would find it useful to have this kind of reminder on the software when a patient enters the recommended ages.

### 3.5. Pharmacist’s Opinion on the Evolution of the Recommendations Concerning HPV Vaccination

**Figure 4:**
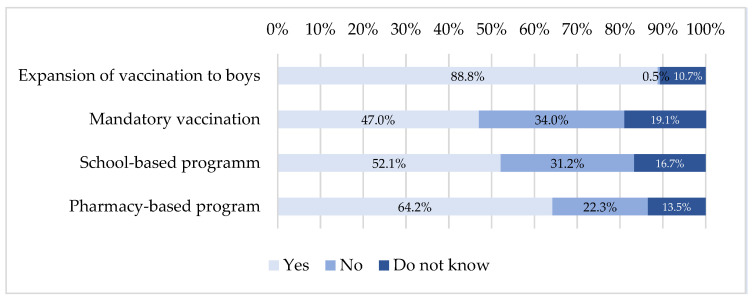
Expansion of recommendations and perspectives.

Figure 4 presents pharmacists’ perspectives on expanding recommendations and prospects for changing HPV vaccination behavior.

The vast majority of respondents felt that expanding the target population to all adolescents, regardless of gender and sexual orientation, was appropriate, at 93.5% (n = 201), and supported expanding HPV vaccination, at 88.8% (n = 191).

Among the pharmacists in favor, 57 responses were entered, the reasons cited were better prevention and therefore protection (57.9%, n = 33), better gender and sexuality equity (10.5%, n = 6), that it protects against serious diseases (17.5%, n = 10), that it is obvious/logical (12.3%, n = 7), and out of caution (1.8%, n = 1).

Concerning the perception of the evolution of this vaccination, 47.0% (n = 101) were in favor of a mandatory vaccination, 52.1% (n = 112) were in favor of a vaccination program in schools, and 64.2% (n = 138) were in favor of a vaccination program directly at the pharmacy.

### 3.6. Determinants of the Level of Knowledge toward HPV and Its Vaccination

**Figure 5:**
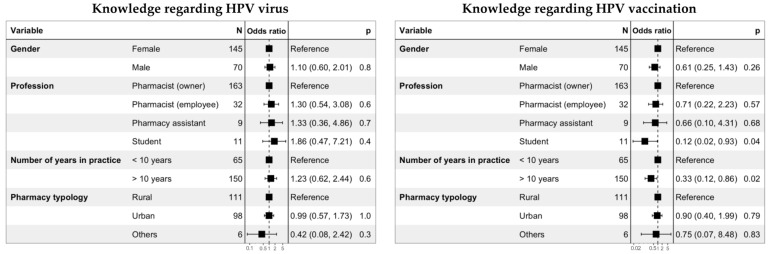
Ordinal logistic regressions performed to evaluate the determinants of the level of knowledge related to HPV and HPV vaccination.

The results of the ordinal logistic regressions performed to evaluate the determinants of a better level of knowledge related to HPV and its vaccination are presented in Figure 5. The gender, the profession, the number of years of practice, and the pharmacy topology were not significantly associated with the level of knowledge about HPV. Regarding vaccination, a lower level of knowledge was significantly associated with students (OR = 0.12 (0.02–0.93); *p* = 0.04) and with professionals with more than 10 years of practice (OR = 0.33 (0.12–0.86); *p* = 0.02).

### 3.7. Determinants of the Attitudes and Practices of Pharmacists regarding HPV Vaccination

#### 3.7.1. Determinants of the Attitudes of Pharmacists Regarding HPV Vaccination in General

**Figure 6:**
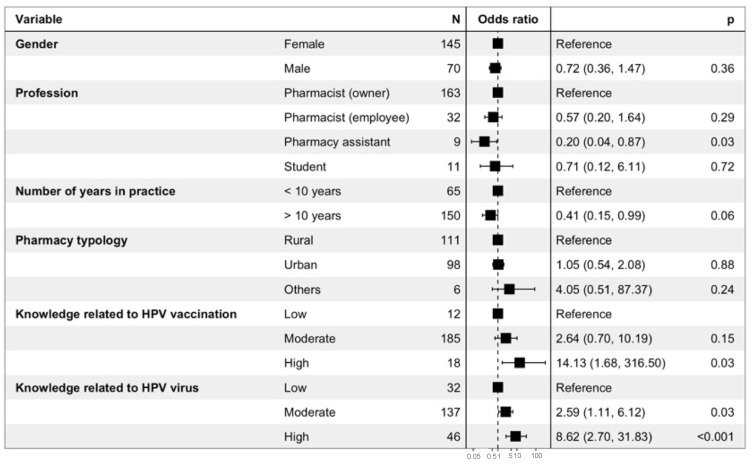
Binary logistic regression performed to evaluate the determinants of the right attitude toward HPV vaccination in general. The right attitude toward HPV vaccination in general corresponded to respondents that (i) were highly or very highly confident in HPV vaccines, (ii) felt comfortable or rather comfortable arguing with questions about vaccines in general, (iii) felt comfortable or rather comfortable arguing with questions about HPV vaccines, (iv) felt concerned about HPV, and (v) found that promoting HPV vaccination is part of pharmacists’ roles.

The results of the binary logistic regression performed to evaluate the determinants of the right attitude toward HPV vaccination in general are presented in Figure 6. Gender, number of years in practice, and pharmacy typology were not significantly associated with a better attitude toward HPV vaccination. Pharmacy assistants were less likely to have the right attitude (OR = 0.20 (0.04–0.87); *p* = 0.03), while a high level of knowledge related to HPV (OR = 8.62 (2.70–31.83); *p* < 0.001) and HPV vaccination (OR = 14.13 (1.68–316.50); *p* = 0.03) were positively related to a better attitude toward vaccination in general.

#### 3.7.2. Determinants of the Attitudes of Pharmacists Regarding HPV Vaccination in Girls

**Figure 7:**
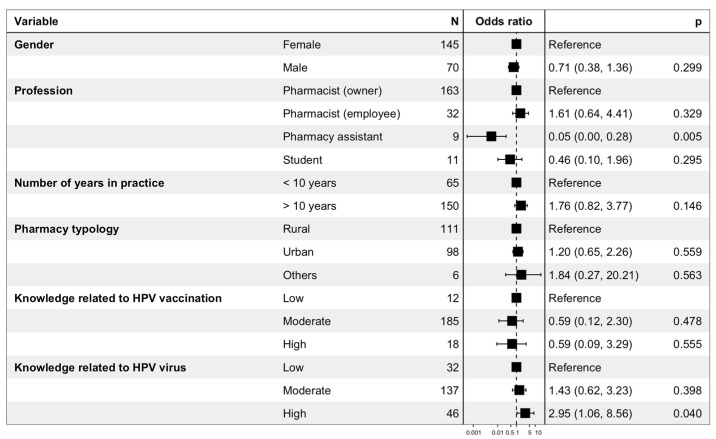
Binary logistic regression performed to evaluate the determinants of the right attitude toward HPV vaccination in girls. The right attitude toward HPV vaccination in girls corresponded to respondents that (i) found HPV vaccine useful for girls, (ii) considered that HPV vaccine was safe for girls, and (iii) had previously advised HPV vaccination to an 11–14-year-old girl.

The results of the binary logistic regression performed to evaluate the determinants of the right attitude toward HPV vaccination in girls are presented in Figure 7. Gender, number of years in practice, pharmacy typology, and level of knowledge related to HPV vaccination were not significantly associated with a better attitude toward HPV vaccination in girls. Pharmacy assistants were less likely to have a good attitude (OR = 0.05 (0.00–0.28); *p* = 0.005), while a high level of knowledge related to HPV was positively related to a better attitude toward vaccination in girls (OR = 2.95 (1.06–8.56); *p* = 0.04).

#### 3.7.3. Determinants of the Attitudes of Pharmacists Regarding HPV Vaccination in Boys

**Figure 8:**
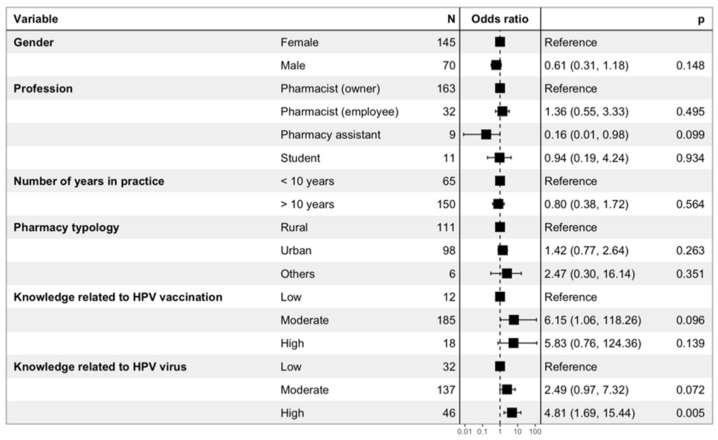
Binary logistic regression performed to evaluate the determinants of the right attitude toward HPV vaccination in boys. The right attitude toward HPV vaccination in boys corresponded to respondents that (i) found HPV vaccine useful for boys, (ii) considered that HPV vaccine was safe for boys, and (iii) had previously advised HPV vaccination to an 11–14-year-old boy.

The results of the binary logistic regression performed to evaluate the determinants of the right attitude toward HPV vaccination in boys are presented in Figure 8. Gender, number of years in practice, and pharmacy typology were not significantly associated with a better attitude toward HPV vaccination in boys. Pharmacy assistants were less likely to have a good attitude (OR = 0.16 (0.01–0.98); *p* = 0.099), while a high level of knowledge related to HPV (OR = 4.81 (1.69–15.44); *p* = 0.005) and a moderate level of knowledge related to HPV vaccination (OR = 6.15 (1.06–118,26); *p* = 0.096) were positively related to a better attitude toward vaccination in boys.

#### 3.7.4. Determinants of the Practices of Pharmacists Regarding HPV Vaccination

**Figure 9:**
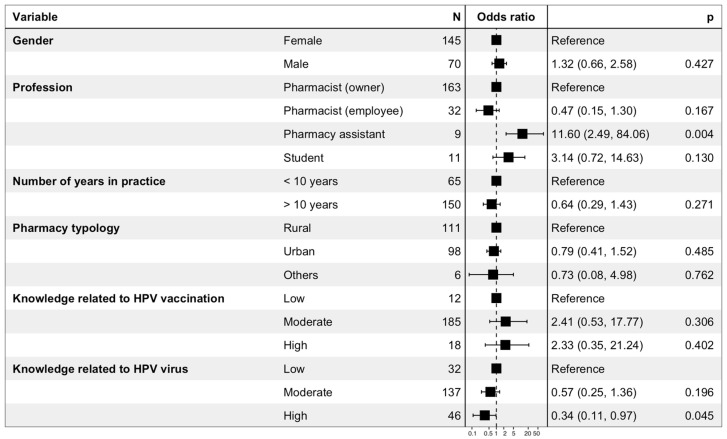
Binary logistic regression performed to evaluate the determinants of never having advised the HPV vaccine to an 11–14-year-old girl, an 11–14-year-old boy, or a man 18–26 years of age who has sex with men.

The results of the binary logistic regression performed to evaluate the determinants of never having advised the vaccine are presented in Figure 9. Gender, number of years in practice, pharmacy typology, and level of knowledge related to HPV vaccination were not significantly associated with a better attitude toward HPV vaccination in boys. Pharmacy assistants were more likely to never have advised the vaccine (OR = 11.6 (2.49–84.06); *p* = 0.004), while a high level of knowledge related to HPV (OR = 0.34 (0.11–0.97); *p* = 0.045) was positively related to having already advised the HPV vaccine.

## 4. Discussion

HPV contamination remains a major public health problem in France. The French population remains under-vaccinated. Only 24% of girls were fully vaccinated in 2018 [26]. The main obstacle to HPV vaccination is the reluctance of children and parents to use this vaccine. HPV vaccination was accepted by 34.4% of boys [54] and 41% of parents of boys aged 11 to 19 years [55] Acceptability of the HPV vaccine is closely related to knowledge of HPV and specifically to perceived risk of HPV infection (in parents and children), a sense of protection from genital warts and cancers, and recognition of vaccine efficacy (in children) [54,56,57,58,59,60]. To increase the acceptability of the HPV vaccine and vaccination, it is necessary that parents and adolescents have a better knowledge of the virus and its complications. Healthcare professionals and, more particularly, pharmacists appear as key persons to reach this goal because the population trusts them [61]. Thus, it is important to better understand the knowledge, attitudes, and practices of pharmacists in order to strengthen their role in the promotion of HPV vaccination.

First, there is a gap between pharmacists’ perceived knowledge and their actual level of knowledge. In our sample, about 50% of the professionals considered their level of knowledge of HPV as “excellent” or “rather excellent”, and this proportion raised to more than 63% about HPV vaccination. However, this was not confirmed by the results obtained from the knowledge questions. Only 21.4% and 8.4% obtained a high level of knowledge related to, respectively, HPV and vaccination. Other studies also demonstrated a lack of knowledge regarding HPV vaccination among health professionals [62,63,64]. In addition, in our study, higher levels of pharmacist knowledge were positively associated with better attitudes toward HPV vaccination in general, among both girls and boys, and a greater likelihood of advising the vaccine. Thus, it is important to improve the knowledge of pharmacists concerning HPV and vaccination.

Secondly, pharmacists were confident in HPV vaccines (94.4%). Similar rates are found among General Practitioners (GPs), 94% of whom are in favor of this vaccination in 2019 [65]. However, this confidence was lower among parents of French adolescents, falling between 50% [60] and 75% [65]. The majority of respondents found the HPV vaccine useful and safe. However, doubts about the usefulness and safety of the vaccine were greater for boys. These same doubts are found among physicians, 12% of whom find the safety data insufficient for young girls and 27% for boys [66]. However, the vaccine GARDASIL 9^®^ has been shown to have the same safety and tolerability profile regardless of gender, as well as efficacy in the prevention of genital warts and HPV-related precancerous and cancerous lesions [67].

Pharmacists appeared to be less comfortable with questions about HPV vaccines than about vaccines in general. This problem was also noted among GPs. HPV vaccination was cited by 82% of them as one of the most difficult vaccinations to get patients and parents to adhere to and convince them of its usefulness [65].

Regarding the perception of their role in promoting vaccination, the vast majority of participants felt concerned by this prevention method and found that promoting this vaccination was part of their role. However, the rate of participants having already been advised this vaccination was low. For GPs, the frequency of vaccine recommendations varied greatly depending on the vaccine and the vaccination status [68]. For HPV vaccination, between 72% [69,70] and 91% [65] of GPs said they always or often offered this vaccination to their patients aged 11 to 14 years. Regarding boys, 84% said they would recommend it if it were part of the vaccination schedule [65], but only 9.9% of MSM had been offered the vaccine by their family physician [71]. However, 88% of GPs who did not routinely recommend vaccination for girls would be more likely to do so if vaccination for boys was also recommended [65].

Thirdly, our study demonstrated that there is a need to increase knowledge of pharmacists and enhance their behavior and practices to recommend HPV vaccination and, more particularly, in boys.

To increase knowledge, training courses could be implemented. Courses have been shown to be effective in improving knowledge, particularly knowledge of the HPV burden in men, and in increasing the comfort of professionals in counseling the HPV vaccine [72,73]. In addition, these courses could be integrated into the continuing education program, which is mandatory for pharmacists throughout their professional practice [74].

To promote HPV vaccination, several solutions can be considered. For the target population, a computerized reminder in the pharmacy software could be added either when the adolescent enters the recommended age range or when the diphtheria–tetanus–polio vaccine reminder is given between 11 and 13 years of age. It could also be a reminder to give a flyer to the patient, for example. Moreover, as the vaccination schedule extends over several months, an alert system could also be set up for dose reminders. Only 4.2% (n = 9) of respondents reported that their software included a reminder for vaccinations, while nearly 90% would find it useful. Interest in automatic recall has also been found among Canadian pharmacists in the follow-up of patients on antidepressants [75]. These types of computerized reminders to physicians have been shown to be effective in the United States in increasing influenza and pneumococcal vaccination rates [76,77,78,79].

Moreover, the implementation of promotional materials could be another element used to promote vaccination. Among the respondents, less than 10% of pharmacists used promotional materials for HPV vaccination, particularly flyers, and only about 25% of them mentioned boys and MSM. Nevertheless, almost 80% of pharmacists would be interested in obtaining such materials. This interest in summary leaflets has also been shown among GPs and pharmacists in New Zealand [80] and Canada [75]. In addition, several studies have demonstrated their effectiveness in increasing the acceptability of HPV vaccination [81,82], Herpes Zoster vaccination [83], or the proper use of antibiotics [84].

Finally, pharmacists (88.8%) were in favor of the expansion of recommendations for vaccinating boys and found it appropriate. The same conclusions were raised by physicians (87% [66]).

Concerning the perspectives of the evolution of recommendations, only about 50% of participants were in favor of a mandatory vaccination or a school-based program. These rates are much lower than those found in other European countries; 77% of gynecologists in Poland [83] and 91% of health professionals in Italy [85] are in favor of mandatory vaccination. In addition, several studies suggest that American [86,87] and Australian parents tend to support school-based vaccination programs for their children [88].

Since 2018, the vaccination obligation for already 11 vaccines in infants [89] has allowed achieving coverage of more than 75% in children [90]. Including HPV vaccination in these vaccines could therefore have a great impact on the rate of vaccinated adolescents.

A school-based vaccination program, as in Australia and Quebec, could increase current vaccination coverage. In 2019, the experimentation of this vaccination in schools was set up in two regions of France for a period of three years [91,92]. The results will allow for the consideration of a national extension.

Additionally, there is a need to increase access to vaccination in France. More than 60% of pharmacists were in favor of a pharmacy-based program. In January 2022, the HAS recommended the extension of vaccination competencies for nurses, midwives, and pharmacists [93]. This new recommendation authorizes them to administer non-live vaccines, including the anti-HPV vaccine, for people aged 16 and over. In addition, in June 2022, the HAS extended its recommendation to all vaccines on the vaccination calendar from the age of two [94]. This simplification of the vaccination process will improve vaccination coverage against HPV. Indeed, pharmacists administered 34% of the 2020–2021 seasonal flu vaccines and 60% of the COVID-19 vaccines

This study has several strengths. Firstly, to our knowledge, this is the first study evaluating the knowledge, behavior, and practices of the pharmacist concerning HPV vaccination in France. Secondly, the respondents are representatives of French pharmacists. The percentage of women among the respondents was the same as that of the French population of pharmacists (68% in 2020) [95], and the topology of pharmacy was similar to the French one [96].

This study has several limitations. First, a limitation is the use of a cross-sectional study that does not allow for analysis of causal relationships and the use of self-reported measures that are susceptible to recall bias and social desirability bias. No comparison between questionnaire responses and actual vaccination practices is possible. Given that, to our knowledge, there is no standardized tool to assess the knowledge, attitudes, and practices concerning HPV of pharmacists in France, we constructed the questionnaire on a theoretical framework established [97,98] following the WHO recommendations [52]. Second, this study was realized in 2021, a period during which the COVID-19 vaccination was part of the pharmacist’s daily routine, which may have influenced certain responses. Third, the sample size (215 respondents) was larger than the minimum required (196 respondents), which allowed for reliable results. Even though the sample size was achieved, the response rate (7.2%) was lower than the response rate described for an online survey offered by e-mail (25–30%) [99]. Moreover, this survey took place when pharmacists were mostly mobilized for screening and COVID-19 vaccination. Pharmacists were contacted twice by e-mail to propose the survey. To increase the response rate, it would have been interesting to send more e-mail reminders and to use a multi-mode approach (e-mail, online questionnaire, postal questionnaire, etc.) [99]. Fourth, the students included in this study represented only 5.1% of the respondents even though they represent the “future” of the pharmacy profession. Indeed, students were not our main target. Thus, it would be interesting, in another study, to include more students and compare their knowledge, attitudes, and practices regarding HPV vaccination with those of pharmacists who have been working for several years. Fifth, it is known that being vaccinated can be a factor in advising it to others. However, given the date of introduction of vaccination recommendations and the duration of pharmacist studies in France, it was not possible to study this factor. Sixth, the number of pharmacy students and pharmacy assistants was low, relative to pharmacists. Therefore, the results of the regression analysis by profession need to be confirmed.

## 5. Conclusions

The results of this study highlight that pharmacists were interested in HPV vaccination and their role as promoters. However, they need resources to facilitate this task, such as training, computer alerts, and supportive materials (e.g., flyers). This will improve their knowledge, help them open discussions with patients, and facilitate vaccination.

It is also important to emphasize that an evolution of the vaccination program, particularly with pharmacy-based vaccination, should facilitate access to vaccination. Vaccination by the pharmacist in addition to prescription would simplify the vaccination process. Indeed, the patient would no longer need to go through different health professionals but could be entirely taken care of at the pharmacy.

## Figures and Tables

**Table 1:** Sociodemographic characteristics of the pharmacists in our sample.

Variable		n (%)
Profession	Pharmacist (owner)	163 (75.8)
	Pharmacist (employee)	32 (14.9)
	Pharmacy assistant	9 (4.2)
	Student	11 (5.1)
Gender	Female	145 (67.4)
	Male	70 (32.6)
Number of years in practice	<5 years	39 (18.1)
	6 to 10 years	26 (12.1)
	11 to 20 years	56 (26.0)
	21 to 30 years	57 (26.5)
	>30 years	37 (17.2)
Pharmacy typology	Urban	98 (45.6)
	Rural	111 (51.6)
	Others ^1^	6 (2.8)

The results are expressed as n (%). n: number of respondents, %: percentage. The total number of respondents was 215. ^1^ Supermarket, Suburban, Ski resort, Shopping center.

**Table 2:** Knowledge of pharmacists related to HPV and HPV vaccination among French pharmacists.

Variable	n (%)
**Self-assessment knowledge**	
HPV knowledge level is excellent or rather excellent	106 (49.3)
Vaccine knowledge level is excellent or rather excellent	136 (63.3)
**Knowledge related to HPV ^a^**	
Low	32 (14.9)
Moderate	137 (63.7)
High	46 (21.4)
Incidence of HPV-induced cancers among men represents between 1500 and 2000 cases/year ^b^	41 (19.1)
HPV infection is the most common sexually transmitted infection ^b^	103 (47.9)
HPVs induce oral sphere cancers among men ^b^	133 (61.9)
HPVs induce penis cancers among men ^b^	131 (60.9)
HPVs induce anus cancers among men ^b^	178 (82.8)
HPVs do not induce testis cancers among men ^b^	145 (67.4)
HPVs do not induce prostate cancers among men ^b^	180 (83.7)
**Knowledge related to HPV vaccination ^c^**	
Low	12 (5.6)
Moderate	185 (86.0)
High	18 (8.4)
In 2019, girls’ vaccination coverage represented between 20 and 30% ^d^	69 (32.1)
The vaccine is recommended for girls between 11 and 14 years old ^d^	214 (99.5)
The vaccine is recommended for boys between 11 and 14 years old ^d^	192 (89.3)
The vaccine is recommended for men who have sex with men until 26 years old ^d^	74 (34.4)

The results are expressed as n (%). n: number of respondents, %: percentage. The total number of respondents was 215. ^a^ Knowledge: Low (0 to 2 right answers), Moderate (3 to 5 right answers), High (6 or 7 right answers). ^b^ Answers considered to determine the level of knowledge related to HPV. ^c^ Knowledge: Low (0 or 1 right answer), Moderate (2 or 3 right answers), High (4 right answers). ^d^ Answers considered to determine the level of knowledge related to HPV vaccination.

**Table 3:** Attitudes and practices of French pharmacists regarding HPV vaccination.

Variable	n (%)
**Attitude and practice regarding HPV vaccination**	
High or very high confident in HPV vaccines	203 (94.4)
Feels comfortable or rather comfortable arguing with questions about vaccines in general	209 (97.2)
Feels comfortable or rather comfortable arguing with questions about HPV vaccines	170 (79.1)
Feels concerned about HPV	202 (94.0)
Find that promoting HPV vaccination is part of pharmacists’ roles	202 (94.0)
**Attitude and practice regarding HPV vaccination in girls**	
HPV vaccine is useful for girls	214 (99.5)
HPV vaccine is safe for girls	198 (92.1)
Previously advised HPV vaccination to an 11–14-year-old girl	150 (69.8)
**Attitude and practice regarding HPV vaccination in boys or young men**	
HPV vaccine is useful for boys	199 (92.6)
HPV vaccine is safe for boys	189 (87.9)
Previously advised HPV vaccination for an 11–14-year-old boy	73 (34.0)
Previously advised HPV Vaccination for men 18–26 years of age who have sex with men	16 (7.4)
Has ever been faced with a prescription for HPV vaccine for a boy or a young man	152 (70.7)
He dispensed it and he knew the condition of reimbursement ^a^	91 (59.9)
He dispensed it after checking the conditions of reimbursement ^a^	47 (30.9)
He dispensed it without checking or knowing the conditions of reimbursement ^a^	14 (9.2)
He refused to dispense it ^a^	0 (0.0)
**Reasons why the pharmacist did not recommend vaccination ^b^**	
Did not have the opportunity	42 (38.9)
Did not think of it	20 (18.5)
Complicated topic to discuss	16 (14.8)
Lack of information/knowledge	16 (14.8)
Recommendation for boys too recent	8 (7.4)
Physician’s role	4 (3.7)
The current battle was the Coronavirus disease 2019 (COVID-19) vaccination	2 (1.9)

The results are expressed as n (%). n: number of respondents, %: percentage. The total number of respondents was 215. ^a^ 152 respondents who have been faced with a prescription for HPV vaccine for a boy or a young man answered this question. ^b^ 108 respondents who have not recommended HPV vaccination answered this question.

## Data Availability

Data are available on request to the corresponding author.

## Supplementary Materials

The following supporting information can be downloaded at https://www.mdpi.com/article/10.3390/v15030778/s1. Table S1: questionnaire regarding knowledge, issues, and role of pharmacists in human papillomavirus vaccination in boys; Table S2: assessment of knowledge of pharmacists regarding HPV and HPV vaccination; and Table S3: assessment of perception and habits of pharmacists regarding HPV vaccination.
