# Supplementary material for: Human Papillomaviruses in Adolescents: Knowledge, Attitudes, and Practices of Pharmacists Regarding Virus and Vaccination in France
Source: Viruses. 2023 Mar 17;15(3):778. doi: 10.3390/v15030778 (PMC10058809; doi:10.3390/v15030778)
Supplement: Supplementary file 1 [file viruses-15-00778-s001.zip › viruses-2277193-supplementary.pdf]

Table S1. Questionnaire regarding knowledge, issues and role of pharmacists in human papillomaviruses vaccination in boys

1) What is your profession?	Pharmacist (owner)<br>Pharmacist (employee)<br>Pharmacy assistant<br>Pharmacy student
2) What is your gender?	Male<br>Female<br>Other
3) What is the typology of the pharmacy?	Rural<br>Semi-rural (5000 to 25000 inhabitants)<br>Downtown<br>Neighborhood
4) In which department is the pharmacy located?	01 – Ain<br>03 – Allier<br>07 – Ardèche<br>15 – Cantal<br>26 – Drôme<br>38 – Isère<br>42 – Loire<br>43 – Haute-Loire<br>63 – Puy-de-Dôme<br>69 – Rhône<br>73 – Savoie<br>74 – Haute-Savoie
5) How long have you been in practice?	< 5 years<br>6 to 10 years<br>11 to 20 years<br>21 to 30 years<br>> 30 years
6) In your opinion, what is your level of knowledge about the HPV virus?	1 2 3 4<br>Insufficient Excellent
7) In your opinion, what is your level of knowledge about HPV vaccination?	1 2 3 4<br>Insufficient Excellent
8) What sources of information do you use to maintain your knowledge? *(open-ended question)*	
9) In your opinion, what is the level of training and information for pharmacists to maintain their knowledge on HPV vaccination?	1 2 3 4<br>Insufficient Excellent
10) In your opinion, what is your patients' level of knowledge about the HPV virus and its vaccine?	1 2 3 4<br>Insufficient Excellent
11) Do you think HPV vaccines are...?<br>11a. Useful for girls<br>11b. Useful for boys<br>11c. Safe for girls<br>11d. Safe for boys	Yes No Don't know

12) In 2021, the vaccine is recommended for: *(several possible choices)*	Girls between 11 and 14 years old, with the possibility of catching up<br>Boys between 11 and 14 years old, with the possibility of catching up<br>Men who have sex with men (MSM) up to age 21<br>Men who have sex with men (MSM) up to age 26<br>Men who have sex with men (MSM) up to age 30
13) What is your estimate of the incidence of HPV-related cancers in men in France?	< 1000 cases per year<br>1000 to 1500 cases per year<br>1500 to 2000 cases per year<br>2000 cases per year
14) What types of HPV cancers are diagnosed in these men?	ENT<br>Penis<br>Anus<br>Testis<br>Prostate<br>Other, specify:
15) The goal for 2019 was 60% vaccination coverage for girls, what do you estimate the coverage to be at the end of 2019? (fully vaccinated)	< 20%<br>20 to 30%<br>30 to 40%<br>40 to 50%<br>> 50%
16) In your opinion, HPV infection is the most common sexually transmitted infection in the world.	True<br>False<br>Do not know
17) In your opinion, wearing a condom is an effective way to protect yourself from the virus.	True<br>False<br>Do not know
18) Are you for or against vaccination in general?	1 2 3 4<br>Against For
19) How much do you estimate your confidence in...?<br>19a. Vaccines in general<br>19b. HPV vaccines	Very low Low High Very high
20) Have you ever been given a prescription for an HPV vaccine for a boy or young man?	Yes<br>No
21) If yes, how did you react to this prescription?	You have dispensed and you knew the conditions of reimbursement<br>You have dispensed, after verifying the conditions for reimbursement<br>You have dispensed, without checking or knowing the conditions of reimbursement<br>You refused to dispense

22) Are you comfortable arguing with questions about vaccination?<br>22a. HPV vaccines<br>22b. Other vaccines	Yes Rather yes Rather no No
23) Does the software you are using have a vaccination reminder built in?<br>23a. HPV vaccines<br>23b. Other vaccines	Yes No
24) Would you find it useful to have a reminder on the software when a patient enters the ages of recommendation?	Yes<br>No<br>Do not know
25) Do you take continuing education courses on a regular basis?	Yes<br>No
26) If yes, have you ever attended any vaccination training?	Yes<br>No
27) If yes, did this training mention...?<br>27a. HPV virus<br>27b. HPV vaccination for girls<br>27c. HPV vaccination for boys<br>27d. HPV vaccination for MSM (Men who have sex with men)	Yes No
28) Do you feel concerned about HPV prevention?	Yes<br>No<br>Do not know
29) If not, why not? *(open-ended question)*	
30) Do you feel that promoting HPV vaccination is one of the roles of the pharmacist?	Yes<br>No<br>Do not know
31) Have you ever advised HPV vaccination to a...?<br>31a. Girl between 11 and 14 years old<br>31b. Boy between 11 and 14 years old<br>31c. MSM aged 18 and 26 years old	Yes No
32) If there is a no, why? *(open-ended question)*	
33) How strongly would you recommend it to a...?<br>33a. Girl between 11 and 14 years old<br>33b. Boy between 11 and 14 years old<br>33c. MSM aged 18 and 26 years old	Strongly discouraged Rather discouraged Rather recommended Strongly recommended
34) Do you feel that expanding the target population to all adolescents, regardless of gender and sexual orientation, was appropriate?	Yes<br>No<br>Do not know
35) Were you in favor of expanding HPV vaccination?	Yes<br>No<br>Do not know
36) Why? *(open-ended question)*	
37) Are you in favor of mandatory vaccination as in some countries?	Yes<br>No

	Do not know
38) Are you in favor of a school-based vaccination program as in some countries?	Yes<br>No<br>Do not know
39) Are you in favor of a pharmacy-based vaccination program?	Yes<br>No<br>Do not know
40) Do you use any materials to promote HPV vaccination?	Yes<br>No<br>Do not know
41) If yes, do they mention vaccination for...?<br>41a. Boys (11-14 years old)<br>41b. MSM	Yes No Don't know
42) What are these materials? *(open-ended question)*	
43) If not, why not?	Lack of information on these materials<br>Lack of time<br>No interest/usefulness<br>No material available<br>Do not feel involved in this promotion/role of physicians<br>Other, specify :
44) Would you be interested in obtaining supports?	Yes<br>No<br>Do not know
45) What strategies/tools/supports could improve this vaccination coverage in your practice? *(open-ended question)*	
46) If you have any comments, any idea to improve the situation or any suggestions, you can specify here: *(open-ended question)*	

Table S2. Assessment of knowledge of pharmacists regarding HPV and HPV vaccination

**Variable**		**N (%)**
HPV VIRUS		
Self-assessment of HPV knowledge level[1]	Excellent or rather excellent	106 (49.3)
	Insufficient or rather insufficient	109 (50.7)
HPV infection is the most common sexually transmitted infection[1]	True	103 (47.9)
	False	34 (15.8)
	Do not know	78 (36.3)
Condom effectively protects against the virus[1]	True	118 (54.9)
	False	66 (30.7)
	Do not know	31 (14.4)
Incidence of HPV-induced cancers among men[1]	< 1000 cases/year	56 (26.0)
	1000 to 15000 cases/year	91 (42.3)
	1500 to 2000 cases/year	41 (19.1)
	> 2000 cases/year	27 (12.6)
Types of HPV-induced cancers among men[2-3]	Oral sphere	133 (61.9)
	Penis	131 (60.9)
	Anus	178 (82.8)
	Testis	70 (32.6)
	Prostate	35 (16.3)
	Genital warts	1 (0.5)
	All the genital area	1 (0.5)
	Do not know	3 (1.4)
HPV VACCINATION		
Self-assessment of HPV vaccine knowledge level[1]	Excellent or rather excellent	136 (63.3)
	Insufficient or rather insufficient	79 (36.7)
The vaccine is recommended for[2] :	Girls between 11 and 14 years old	214 (99.5)
	Boys between 11 and 14 years old	192 (89.3)
	MSM* until 21 y/o	49 (22.8)
	MSM* until 26 y/o	74 (34.4)
	MSM* until 30 y/o	11 (5.1)
Vaccination coverage (in 2019)[1]	< 20%	35 (16.3)
	20 to 30%	69 (32.1)
	30 to 40%	66 (30.7)
	40 to 50%	35 (16.3)
	> 50%	10 (4.7)

Table S3. Assessment of perception and habits of pharmacists regarding HPV vaccination.

Variable		N (%)
Confident in HPV vaccines[1]	High or very high	203 (94.4)
	Low or very low	12 (5.6)
Useful and safety[1]	Yes, useful for girls	214 (99.5)
	Yes, useful for boys	199 (92.6)
	Yes, safe for girls	198 (92.1)
	Yes, safe for boys	189 (87.9)
Feels concerned about HPV[1]	Yes	202 (94.0)
	No	2 (0.9)
	Do not know	11 (5.1)
Find that promoting HPV vaccination is part of them roles[1]	Yes	202 (94.0)
	No	7 (3.3)
	Do not know	6 (2.8)
Ever advised HPV vaccination to a patient[2]	Yes, to an 11-14 y/o girl	150 (69.8)
	Yes, to an 11-14 y/o boy	73 (34.0)
	Yes, to an 18-26 y/o MSM	16 (7.4)
Why not?[3] (n=108)	Did not have the opportunity	42 (38.9)
	Did not think of it	20 (18.5)
	Complicated topic to discuss	16 (14.8)
	Lack of information/knowledge	16 (14.8)
	Recommendation for boys too recent	8 (7.4)
	Physician's role	4 (3.7)
	The current battle was the covid-19 vaccination	2 (1.9)
Ever been faced with a prescription for a HPV vaccine for a boy or a young man[1]	Yes	152 (70.7)
	No	63 (29.3)
Reaction during this dispensation[1]	I dispensed it and I knew the condition of reimbursement	91 (59.9)
	I dispensed it after checking the conditions of reimbursement	47 (30.9)
	I dispensed it without checking or knowing the conditions of reimbursement	14 (9.2)
	I refused to dispense it	0 (0.0)
Feels comfortable arguing with questions about vaccine in general[1]	Yes or rather yes	209 (97.2)
	No or rather no	6 (2.8)
Feels comfortable arguing with questions about HPV vaccine[1]	Yes or rather yes	170 (79.1)
	No or rather no	45 (20.9)
